# Endoscopic Removal of a Toothpick Penetrating the Gastric Wall and Extending Into the Head of the Pancreas: A Case Report and Brief Literature Review

**DOI:** 10.1002/deo2.70418

**Published:** 2026-08-28

**Authors:** Ken Nakayama, Takanori Ueda, Noriyuki Isohata, Tadashi Kondo, Yuji Hiraoka, Yoshihiro Tanabe, Hironobu Yamazaki, Keiichiro Yoshikata, Masayoshi Yamamoto, Tatsuya Oda

**Affiliations:** ^1^ Department of Surgery Ibaraki Western Medical Center Ibaraki Japan; ^2^ Department of Gastrointestinal and Hepatobiliary–Pancreatic Surgery Faculty of Medicine University of Tsukuba Ibaraki Japan; ^3^ Department of Internal Medicine Ibaraki Western Medical Center Ibaraki Japan; ^4^ Department of Surgery Jichi Medical University Saitama Medical Center Saitama Japan; ^5^ Department of Gastroenterology Nippon Medical School Graduate School of Medicine Tokyo Japan; ^6^ Department of Gastrointestinal and Hepatobiliary–Pancreatic Surgery University Hospital of Tsukuba Ibaraki Japan

**Keywords:** digestive system, endoscopy, foreign bodies, pancreas, stomach

## Abstract

Ingested toothpicks can perforate the gastrointestinal tract and penetrate adjacent organs. Pancreatic involvement is rare, and its management varies between surgical and endoscopic interventions. We report the successful endoscopic removal of a toothpick that penetrated the gastric wall and extended into the head of the pancreas. A 59‐year‐old man presented with a 5‐day history of fever and epigastric pain. Laboratory tests revealed inflammatory changes, with normal serum amylase and hemoglobin levels. Non‐contrast computed tomography (CT) with multiplanar reconstruction (MPR) revealed a linear, slightly hyperattenuating foreign body measuring at least 5 cm, extending from the posterior wall of the lower body of the stomach to the head of the pancreas, with minimal free air, surrounding fat stranding, and no dilation of the main pancreatic duct. Contrast‐enhanced CT revealed no major vascular involvement or active extravasation. Esophagogastroduodenoscopy was performed on hospital day 3 with surgical backup. A wooden foreign body protruding from the gastric mucosa was removed with biopsy forceps through an overtube under conscious sedation. The extracted object was a 6.5‐cm wooden toothpick. The post‐procedural course was uneventful. This case highlights the value of MPR and contrast‐enhanced CT in defining a foreign body's trajectory and determining procedural feasibility. Endoscopic removal may be a safe and minimally invasive alternative to surgery in selected cases of pancreatic penetration.

## 1 Introduction

More than 80% of ingested foreign bodies pass spontaneously [1]. However, sharp objects such as toothpicks pose a substantial risk of perforation and may migrate into adjacent organs, including the liver and pancreas, causing abscess formation, acute pancreatitis, or even fatal hemorrhage. Early diagnosis is challenging because wooden toothpicks are radiolucent and often overlooked, and many patients do not recall ingestion. To our knowledge, only five cases of toothpick penetration into the pancreas have been reported (Table 1) [2, 3, 4, 5, 6]; management comprises endoscopic or surgical removal, depending on imaging findings. Here we report a toothpick that penetrated the gastric wall into the head of the pancreas and was removed endoscopically after detailed preprocedural imaging, thereby avoiding surgery.

**TABLE 1:** Summary of reported cases of ingested toothpicks penetrating the upper gastrointestinal tract and involving the pancreas.

Author (year)	Age/Sex	Symptom duration	Perforation site	Serum amylase (U/L)	Diagnostic modalities	Treatment
Kim et al. (2004) [2]	39/M	5 months	Duodenum	195	EGD, EUS, CT, MRA, and ERCP	Coil embolization and laparotomy
Choi et al. (2016) [3]	74/M	1 month	Duodenum	Not reported	CE‐CT, EUS, and IOUS	Laparotomy
Yu et al. (2022) [4]	42/M	1 month	Stomach (antrum)	398	EGD, EUS, and CT	Laparotomy
Wei et al. (2024) [5]	39/M	12 days	Stomach (upper lesser curvature)	772.4	EGD, EUS, and CT	Endoscopic removal
Li et al. (2025) [6]	37/M	3 weeks	Small intestine	Not reported	CE‐CT, EUS, and enteroscopy	Enteroscopic removal and clip closure
Present case	59/M	5 days	Stomach (lower body)	90	CT, CE‐CT, and EGD	Endoscopic removal

Note: All foreign bodies were wooden toothpicks.

Abbreviations: CE‐CT, contrast‐enhanced computed tomography; CT, computed tomography; EGD, esophagogastroduodenoscopy; ERCP, endoscopic retrograde cholangiopancreatography; EUS, endoscopic ultrasonography; IOUS, intraoperative ultrasonography; M, male; MRA, magnetic resonance angiography.

## 2 Case Report

A 59‐year‐old man presented with a 5‐day history of fever and epigastric pain. His medical history included type 2 diabetes mellitus, hypertension, and atrial fibrillation. He was treated with catheter ablation 5 years earlier and was taking rivaroxaban and metformin; the last dose of rivaroxaban was taken on the morning of presentation. The temperature was 38.3°C, with mild epigastric tenderness and no peritoneal signs. Laboratory tests showed inflammation (white blood cells, 13,400/µL; C‐reactive protein, 16.3 mg/dL). Serum amylase levels remained within the normal range (90 U/L at presentation and 70 U/L on hospital day 3); lipase was not measured, and the hemoglobin was 14.3 g/dL. Two sets of blood cultures obtained on admission showed no growth.

**FIGURE 1:**
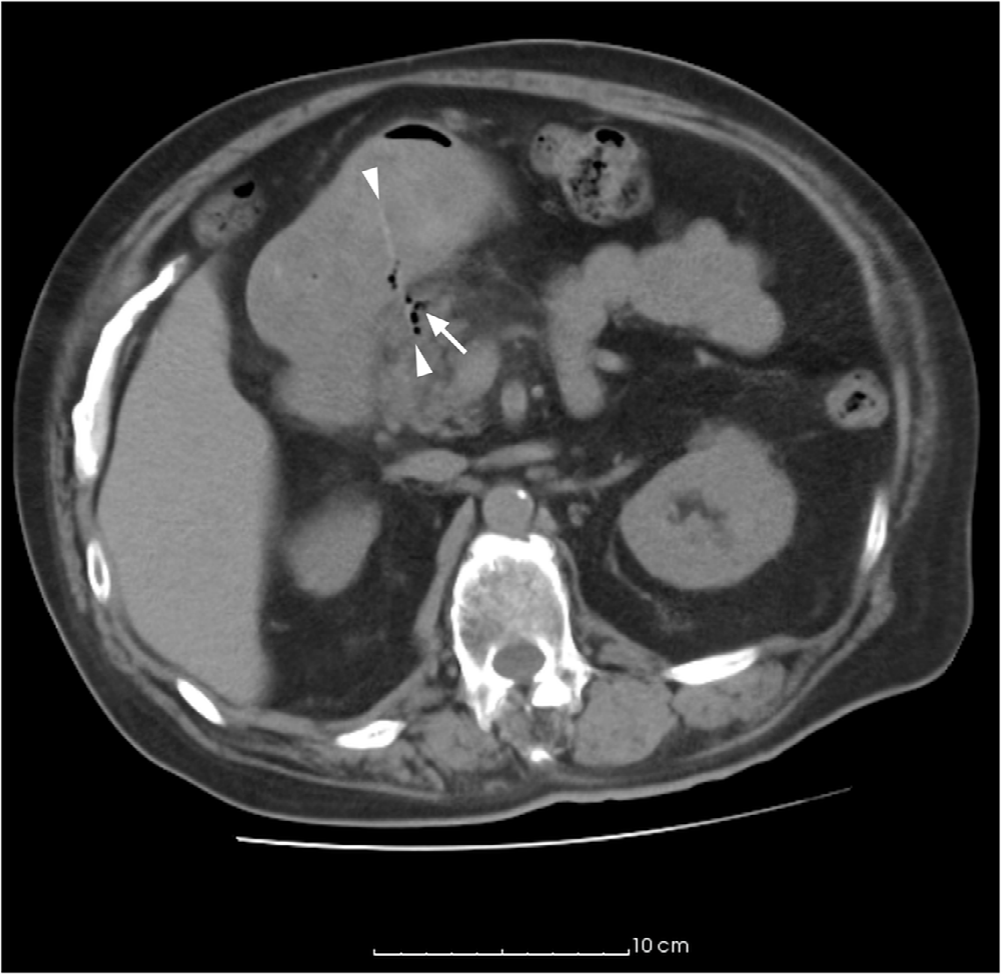
Non‐contrast computed tomography (CT) image obtained at presentation, showing a linear foreign body penetrating from the posterior wall of the lower body of the stomach into the head of the pancreas. The two arrowheads indicate the ends of the foreign body, and the arrow indicates the free air adjacent to it. Inflammatory fat stranding is seen around the foreign body. Window width, 350 HU; window level, 30 HU.

Non‐contrast computed tomography (CT) with multiplanar reconstruction (MPR), reviewed on a soft‐tissue window (window width, 350 HU; window level, 30 HU), revealed a linear, slightly hyperattenuating foreign body (60–70 Hounsfield units) penetrating the posterior wall of the lower body of the stomach into the head of the pancreas, with a small amount of free air and inflammatory fat stranding (Figure 1). The main pancreatic duct was not dilated (approximately 1 mm), and no peripancreatic collection was identified. Although he did not recall ingesting anything, these findings suggested perforation by a foreign body, most likely a toothpick. The patient was admitted and treated with fasting, nasogastric decompression, intravenous fluids, omeprazole, and piperacillin/tazobactam. All oral medications were withheld on admission, including rivaroxaban; given the bleeding risk of the planned removal and a CHA2DS2‐VASc score of 2, periprocedural bleeding risk was prioritized over uninterrupted anticoagulation. He remained in sinus rhythm throughout the admission.

**FIGURE 2:**
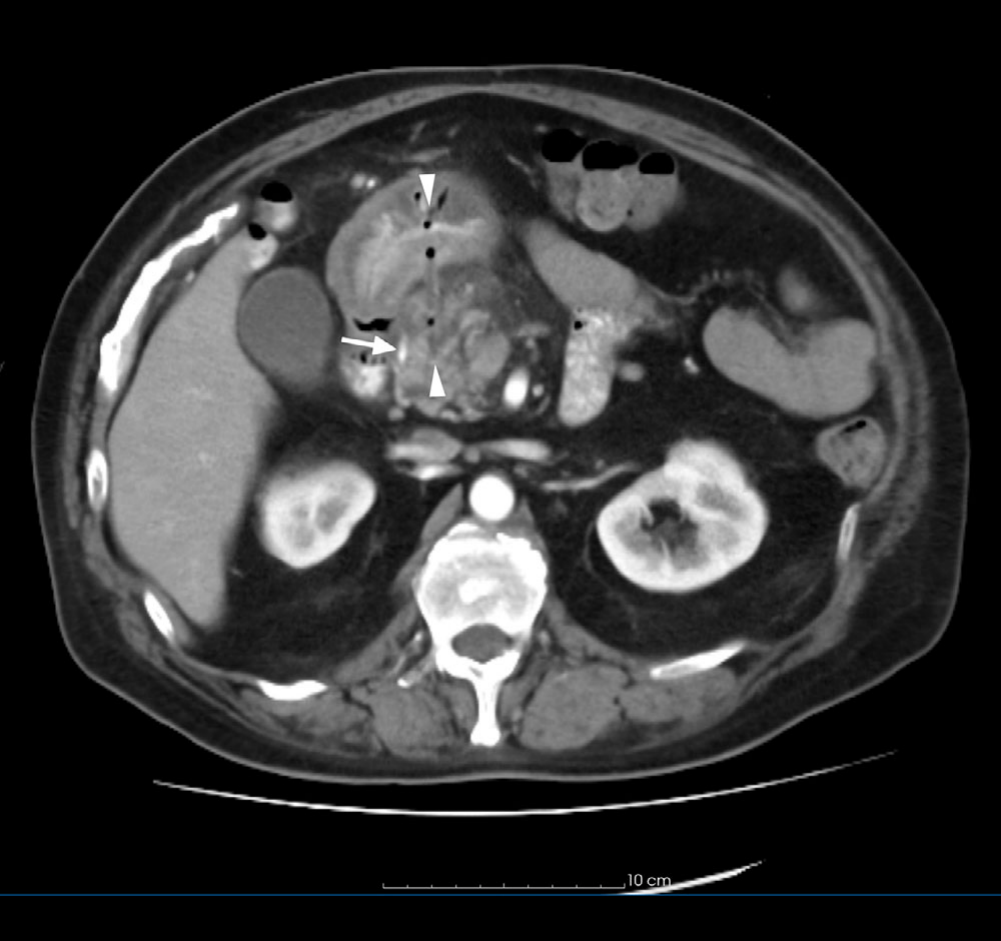
Contrast‐enhanced computed tomography (CT) performed on hospital day 3 for preprocedural vascular assessment, showing that the position of the foreign body was unchanged from the initial study. The two arrowheads indicate the ends of the foreign body, and the arrow indicates the gastroduodenal artery. Although the object lay close to this artery, there was no contact with the celiac artery, superior mesenteric artery, portal vein, or other major vessels, and no active extravasation was seen. Window width, 350 HU; window level, 50 HU. The non‐contrast (Figure 1) and contrast‐enhanced (Figure 2) studies were obtained at different time points and for different purposes—initial diagnosis and preprocedural vascular assessment, respectively—and are therefore presented as separate figures.

On hospital day 3, contrast‐enhanced CT (window width, 350 HU; window level, 50 HU) showed that, although deeply embedded in the head of the pancreas, the object did not contact the celiac artery, superior mesenteric artery, portal vein, or other major vessels, and no active extravasation was observed (Figure 2). As he was hemodynamically stable and responded to conservative management, emergency intervention was not indicated; removal was scheduled once vascular involvement had been excluded, rivaroxaban was withheld for approximately 2 days, and surgical backup was available.

**FIGURE 3:**
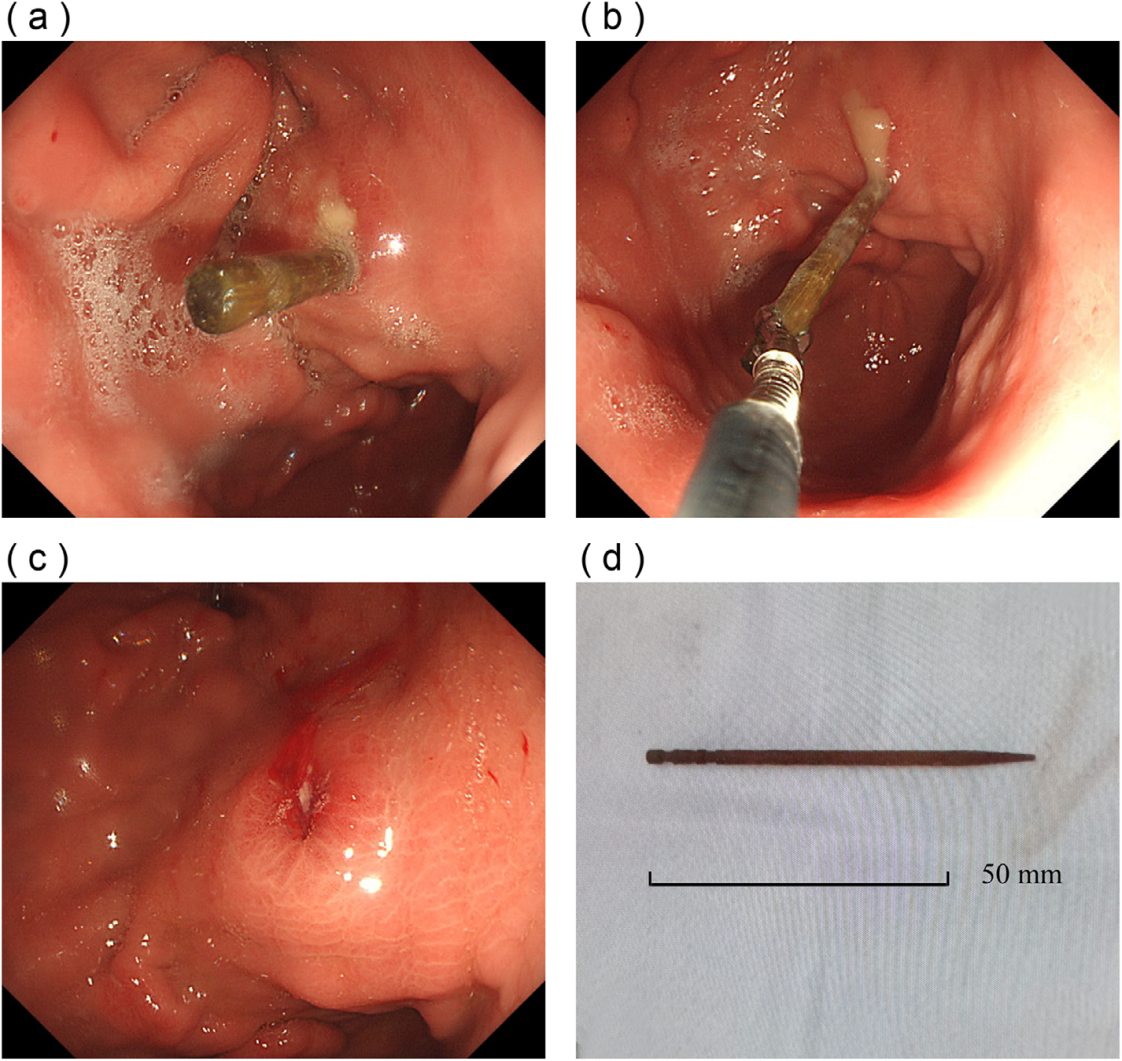
Endoscopic findings and the removed object. (a) A wooden foreign body protruding approximately 1 cm from the mucosa of the posterior wall of the lower body of the stomach. (b) The exposed end was grasped with biopsy forceps and withdrawn along the axis of penetration; a small amount of purulent discharge was noted at the penetration site. (c) The penetration site after thrombin spraying, with hemostasis achieved. (d) The extracted 6.5‐cm wooden toothpick (scale bar, 50 mm).

Esophagogastroduodenoscopy was performed using an overtube under conscious sedation with midazolam. A wooden foreign body protruded approximately 1 cm from the erythematous, edematous mucosa of the posterior wall of the lower body of the stomach (Figure 3a). Its exposed end was grasped with biopsy forceps and withdrawn along the axis of penetration (Figure 3b); a small amount of purulent discharge was noted at the penetration site, from which no specimen was submitted for culture. Mild bleeding was controlled with thrombin spraying (Figure 3c). Because the tract was narrow and walled off, with only localized free air and no diffuse peritonitis, and hemostasis was achieved without ongoing leakage, closure with clips or an over‐the‐scope clip was considered unnecessary. The extracted object was a 6.5‐cm wooden toothpick, whereas CT demonstrated at least 5 cm (Figure 3d).

Follow‐up CT showed no increase in free air and no new fluid collection; as no drainable collection was present, additional drainage was considered unnecessary. The clinical course was uneventful. Oral intake was initiated on hospital day 8, and rivaroxaban was resumed on the 7th day after removal (9 days after admission). The patient was discharged without complications on hospital day 13. Upon repeated questioning, he recalled that he might have fallen asleep while intoxicated with a toothpick in his mouth.

## 3 Discussion

In Steinbach et al.’s review of 136 cases, more than half of the patients were unaware that they had swallowed a toothpick, as in our patient, and the CT sensitivity was approximately 43% [7]. This mainly reflects the attenuation of wood, which is low and variable and frequently overlaps with that of surrounding soft tissue, luminal contents, and gas; therefore, a distinct density contrast is often lacking. In our case, the toothpick was atypically hyperattenuating (60–70 HU) and was identified on a standard soft‐tissue window; thin‐section MPR was the most useful adjunct, allowing its linear course to be traced across successive planes. Therefore, a routine MPR review of soft‐tissue windows whenever a wooden foreign body is suspected is recommended.

PubMed (January 1, 1900, to December 31, 2025) was searched using (toothpick OR “Foreign Bodies” [Mesh]) AND stomach AND (penetration OR perforation) AND pancreas. This retrieved reports of other sharp foreign bodies, such as fish bones and needles, but only one toothpick reaching the pancreas from the stomach [4]; screening the “Similar articles” for that report and the reference lists of retrieved articles identified five cases (Table 1) [2, 3, 4, 5, 6].

Clinical presentations vary widely (Table 1), from bleeding from a splenic artery pseudoaneurysm [2] and an inflammatory mass mimicking pancreatic cancer [3] to acute pancreatitis [4, 5] and indolent abdominal pain [6]. Our patient had normal serum amylase levels and no pancreatic ductal dilation, suggesting localized pancreatic irritation without clinically overt pancreatitis. Lipase, which is more sensitive and specific than amylase for pancreatic injury, was not measured—a limitation of our report—and we recommend assessing both enzymes when pancreatic penetration is suspected.

In all five previous cases, endoscopic ultrasonography (EUS) contributed to the diagnosis, including the two managed without laparotomy [2, 3, 4, 5, 6]. Conversely, our diagnosis, penetration trajectory, and decision to proceed endoscopically relied solely on CT; therefore, the novelty lies in this combination of CT‐based diagnosis and endoscopic removal without EUS, rather than in endoscopic retrieval itself. The site of penetration is anatomically plausible because the posterior wall of the lower body and antrum of the stomach lies in close apposition to the pancreas across the lesser sac. Therefore, a sharp object impacted there and propelled by peristalsis can perforate the head of the pancreas. In previous reports, the entry site varied among the stomach, duodenum, and small intestine (Table 1) [2, 3, 4, 5, 6].

Penetration into a solid organ has traditionally been managed surgically; however, recent reports indicate that endoscopic removal is safe for selected patients [5, 6]. Endoscopic treatment may be considered a first‐line option when part of the foreign body is exposed within the lumen and thus graspable, major vascular injury has been excluded by contrast‐enhanced CT, and the patient is hemodynamically stable without generalized peritonitis or uncontrolled sepsis. The penetration site was not closed, and follow‐up CT confirmed that mechanical closure was not required. Nevertheless, closure devices should be immediately available and used if a large mucosal defect, ongoing leakage, or uncontrolled bleeding is encountered. Although rivaroxaban raised concerns regarding bleeding, exclusion of vascular involvement and the 2‐day withholding period allowed safe removal with surgical backup. Endoscopic management is less invasive than surgery and may avoid complications, such as pancreatic fistula. Notably, the extracted toothpick measured 6.5 cm, whereas CT suggested approximately 5 cm, reflecting the difficulty in precisely delineating a wooden foreign body even with MPR.

In conclusion, endoscopic removal can be an effective and minimally invasive alternative to surgery for toothpick penetration into the pancreas when multiplanar and contrast‐enhanced CT show that the foreign body is endoscopically accessible and not associated with major vascular involvement.

## Author Contributions

**Tadashi Kondo**: writing – review and editing and validation. **Tatsuya Oda**: writing – review and editing and validation. **Yoshihiro Tanabe**: writing – review and editing and validation. **Noriyuki Isohata**: writing – review and editing and validation. **Keiichiro Yoshikata**: writing – review and editing and validation. **Yuji Hiraoka**: writing – review and editing and validation. **Masayoshi Yamamoto**: writing – review and editing and validation. **Ken Nakayama**: writing – original draft, validation, conceptualization, investigation, methodology, visualization, project administration, and data curation. **Takanori Ueda**: writing – review and editing and validation. **Hironobu Yamazaki**: writing – review and editing and validation. All authors approved the final version of the manuscript.

## Funding

The authors have nothing to report.

## Ethics Statement

All the procedures were performed in accordance with the ethical standards of the Declaration of Helsinki and its amendments.

## Consent

Informed consent for this case report was obtained from the patient.

## Conflicts of Interest

The authors declare no conflicts of interest.
